# Chemo-Electrical Gas Sensors Based on Conducting Polymer Hybrids

**DOI:** 10.3390/polym9050155

**Published:** 2017-04-26

**Authors:** Seon Joo Park, Chul Soon Park, Hyeonseok Yoon

**Affiliations:** 1 Hazards Monitoring Bionano Research Center, Korea Research Institute of Bioscience and Biotechnology (KRIBB), 125 Gwahak-ro, Yuseong-gu, 34141 Daejeon, Korea; seonjoopark86@gmail.com (S.J.P.); log71944@gmail.com (C.S.P.); 2 Department of Polymer Engineering, Graduate School, Chonnam National University, 77 Yongbong-ro, Buk-gu, 61186 Gwangju, Korea; 3 School of Polymer Science and Engineering, Chonnam National University, 77 Yongbong-ro, Buk-gu, 61186 Gwangju, Korea

**Keywords:** conducting polymers, composites, sensors, polypyrrole, polyaniline, polythiophene

## Abstract

Conducting polymer (CP) hybrids, which combine CPs with heterogeneous species, have shown strong potential as electrical transducers in chemosensors. The charge transport properties of CPs are based on chemical redox reactions and provide various chemo-electrical signal transduction mechanisms. Combining CPs with other functional materials has provided opportunities to tailor their major morphological and physicochemical properties, often resulting in enhanced sensing performance. The hybrids can provide an enlarged effective surface area for enhanced interaction and chemical specificity to target analytes via a new signal transduction mechanism. Here, we review a selection of important CPs, including polyaniline, polypyrrole, polythiophene and their derivatives, to fabricate versatile organic and inorganic hybrid materials and their chemo-electrical sensing performance. We focus on what benefits can be achieved through material hybridization in the sensing application. Moreover, state-of-the-art trends in technologies of CP hybrid sensors are discussed, as are limitations and challenges.

## 1. Introduction

Many candidate materials have been explored for chemosensing materials, and, accordingly, many different transduction mechanisms have been developed [1]. Conducting polymers (CPs) have been studied extensively since their discovery in 1977 [2]. In 2000, Heeger, MacDiarmid, and Shirakawa jointly received the Nobel Prize in Chemistry, which reignited considerable interest in CP research. At the beginning of 2000, so-called nanoscience and nanotechnology were also hot topics in research worldwide. Many unique and surprising properties of materials were found at the nanoscale. Naturally, various CP nanomaterials have been developed and have novel and fascinating properties [3]. CPs are a new class of electronic materials and are completely different from most existing inorganic electronic materials. Simply, CPs have the advantages of tunable electrical/electronic properties, easy synthesis, structural diversity, flexibility, and environmental stability (e.g., non-corrosive) [4,5,6]. These advantages allow CPs to be explored as alternatives to the inorganic electronic materials that are used in diverse application fields. Of course, CPs have intrinsic limitations, such as a relatively low conductivity and a lack of consistency in properties. Therefore, there has been considerable effort to couple CPs with other heterogeneous species, which has resulted in many remarkable research findings [7,8,9,10]. Regarding sensor applications, the hybridization of CPs with other components has been conducted to improve the sensitivity and selectivity [3,4]. For example, CPs can achieve sensitive responses to various external stimuli, including chemical species, at relatively low temperatures, which corresponds to one of the greatest advantages of using CPs as the sensing material [11,12]. However, unfortunately, the sensitive signal transduction of CPs is available for all external stimuli, not just a target species. Thus, it is not easy to achieve selective, specific responses to a target species using only pure CPs. To overcome this limitation, the chemical structure of CPs can be modified or functional heterogeneous materials can be coupled with CPs. A variety of heterogeneous materials, including quantum dots, two-dimensional inorganic nanomaterials, and nanocarbons (e.g., fullerene, carbon nanotubes, graphene), have been combined with CPs to achieve synergistic effects [7,8,9,10].

**Figure 1:**
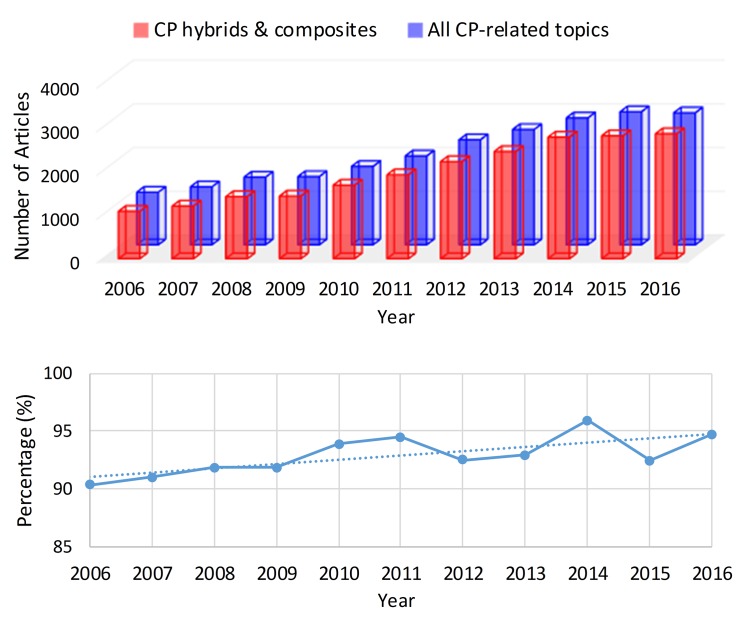
The proportion of articles published on hybrids/composites in the body of CP-related research over the last decade. Data from the ISI Web of Knowledge database.

Figure 1 shows the latest research trend in CPs. CPs have attracted increasing interest over the last decade, and the proportion of hybrids or composites in CP research has continuously increased. The improved properties of the CP hybrids and composites have made it possible to fabricate new, high-performance sensors. Many efforts have been devoted to the development of various kinds of sensors with different transduction modes using the hybrids and composites [13,14]. In this review, we cover the electrical transduction of CP hybrids to detect gas-phase chemical species, and discuss the transduction mechanisms according to the type of hybrid. Several comprehensive review articles have been published regarding CP-based sensors [12,13,14]. Therefore, we focus on what benefits can be obtained in using CP hybrids in chemo-electrical sensor applications. In addition, examples are introduced to present the recent research trends, most of which have been conducted since 2012.

## 2. CP Chemosensors

**Figure 2:**
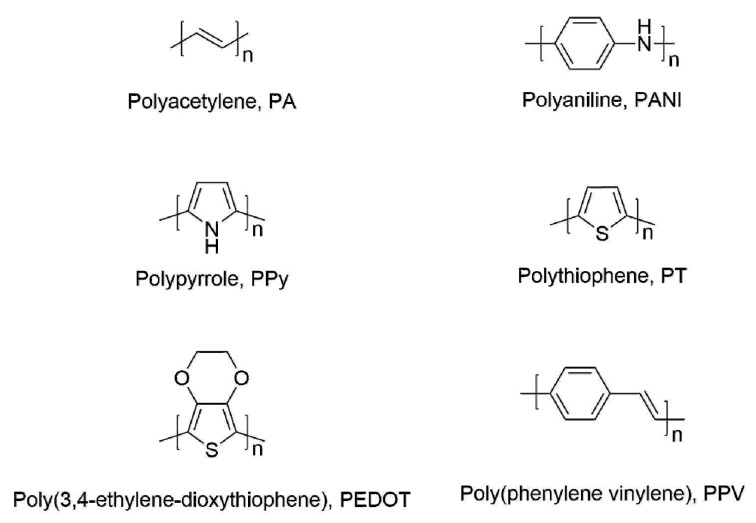
Chemical structures of representative CPs.

CPs, including polypyrrole (PPy), polyaniline (PANI), polythiophene (PTh), poly(3,4-ethylenedioxythiophene (PEDOT), and their derivatives, as shown in Figure 2, have received increasing attention because of their easy fabrication and inherent physical properties, biocompatibility, and optical properties. Their main structures consist of single and double bonds, which are conjugated π–electron systems. Although these conjugated polymer chains have conductivity, their values are very low. To increase their conductivity, a doping process by redox reaction or protonation is needed to remove the electrons on the backbone of the CPs. Positive charges in a CP backbone are accomplished as a charge carrier, leading to conducting properties (conductivity, ca. 10^0^ to 10^5^ S·cm^–1^) [4,13]. In comparison, copper has a conductivity of approximately 10^6^ S·cm^–1^ at room temperature. The unique electrical properties of doped CPs enable energy and electrical applications [4,15,16,17]. In particular, CPs have been used as the active layers in gas-sensing systems owing to their excellent electrochemical properties with target analysis. Compared to conventional chemical sensors using metal oxides or other inorganic materials [18,19,20,21,22,23,24,25], CP-based chemosensors offer attractive advantages such as room temperature operation [26,27,28], facile functionalization [29,30,31], structural diversity [32,33], and easy fabrication [34].

CP chemo-electrical sensors function by analyzing physical or electrochemical signals such as adsorption of gas and charge transfer between gas molecules and CP backbones. Based on the sensing mechanisms, the sensing performance, such as sensitivity and selectivity, of the chemosensors can be improved by controlling the charge transfer behavior between gas molecules and CPs. Yoon et al. [35] demonstrated charge transport behavior between nanostructured PEDOT and chemical gases, including ethanol and acetonitrile, by intrinsic and extrinsic factors. The intrinsic factors (e.g., oxidation level) are associated with the charge transport between the inter- or intra-chains in a single nanoparticle, and the extrinsic factors (e.g., contact resistance) originate from the interaction between nanoparticle–nanoparticle or nanoparticle–metal electrodes. The performance of PEDOT sensors was highly dependent on the two factors. Kwon et al. [36] reported the preparation method of the PEDOT membrane by vapor deposition polymerization. The PEDOT membranes were used for ammonia gas sensors because, having a hole carrier, they easily underwent a redox reaction with electron-donating molecules such as NH_3_. The conductivity of the PEDOT membrane increased with the increasing thickness of the PEDOT membrane, leading to high sensitivity for NH_3_ monitoring. In addition, the same group demonstrated the effect of functional groups on the selectivity of CP sensors. The functional groups can make CPs chemically specific to target species via intermolecular interactions such as hydrogen bonding. For example, the carboxyl group (−COOH) of the PPy backbone can interact with phosphoryl groups of dimethyl methylphosphonate (DMMP) gas, which is similar to nerve agent gases. This interaction changes the charge distribution in PPy, resulting in the decreased movement of the charge carrier holes along the polymer chain. The resistance of the PPy increased with increasing interactions between PPy and DMMP. However, although such advantages of CP transducers have improved the sensing performance of chemosensors, their limitations such as low sensitivity and selectivity still remain a challenge. Recently, inorganic or organic metal oxide/CP hybrid materials have been applied in high-performance chemosensors owing to their synergetic effects and enhanced capacity for gas interaction [1,10,13,14]. In this review, we provide an overview of the state-of-the-art technologies of CP hybrids for gas chemo-electrical sensors and discuss their limitations and challenges.

## 3. CP Hybrids

### 3.1. Inorganic-CP Hybrids

#### 3.1.1. Metal Oxide Hybrids

Metal oxide semiconducting sensors have been widely developed and applied for the detection of chemical molecules because of their structural stability, facile integration, cost efficiency and sensing of most toxic gases [18,19,20,21,22,23]. However, metal oxide semiconducting sensors have a lack of flexibility and selectivity and must be operated at high temperatures (more than 200 °C), causing high power consumption, safety hazards, and short lifetimes [24,25]. By contrast, CP-based sensors have attracted enormous interest because CPs have advantages such as easy synthesis, environmental stability, and sensitive response to chemical molecules at room temperature; however, single CPs exhibit relatively low sensitivity to chemicals. Accordingly, a possible strategy is to combine a CP with a metal oxide, which achieves great sensing performance of the novel sensing material over the constituent counterparts. 

**Figure 3:**
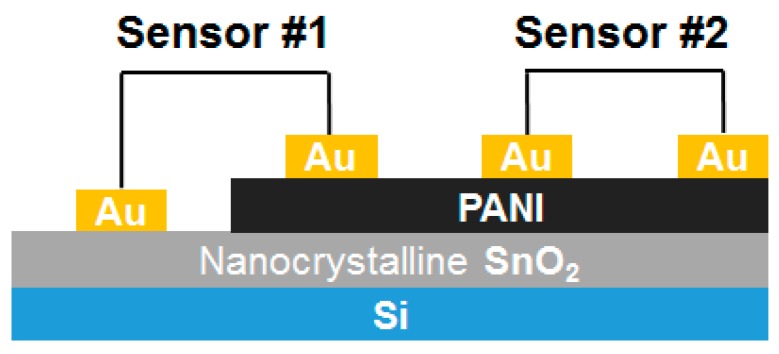
Schematic of the Si/SnO_2_-PANI heterostructure gas sensor with gold contacts [40].

Recently, Quan et al. [37] presented a network structure of a PANI/SnO_2_ hybrid that was synthesized by chemical oxidative polymerization and utilized ZnO nanorods as a template; the hybrid was loaded on a polyethylene terephthalate (PET) film to form a flexible sensor. The sensor exhibited sensitivity 20 times higher than pure PANI to 10 ppm trimethylamine (TEA), great selectivity and a linear response at room temperature. In addition, the SnO_2_/PANI hybrid sensor has flexible, structurally simple, economical, and portable characteristics. Bai et al. [38] reported a MoO_3_/PANI hybrid loaded on a PET film, which was synthesized by an in situ chemical oxidation polymerization method and attained a good linear response to TEA concentrations from 10 to 100 ppm at room temperature. Sen et al. [39] demonstrated carbon monoxide (CO) gas detection using Co_3_O_4_/PANI nanocomposites at room temperature. The Co_3_O_4_ nanoparticles were prepared by an ultrasound-supported coprecipitation technique and then incorporated into the PANI matrix. The sensor exhibited high selectivity to CO gas at room temperature and obtained a significantly high response to 75 ppm CO gas concentration for 40 s. However, the sensing response toward CO gas by the sensor was affected by humidity. A significant decrease (approximately 50%) in response intensity was observed for increased humidity level. Betty et al. [40] proposed sensing the trace amounts of toxic NO_2_ gas at room temperature based on SnO_2_/PANI heterostructure nanocomposites, which were synthesized by the electrochemical deposition of PANI of various thicknesses on nanocrystalline SnO_2_ prepared by the Langmuir-Blodgett method. Two types of sensors were prepared with in-plane gold: sensor #1 contacted one each of SnO_2_ and PANI, and sensor #2 contacted the PANI film (Figure 3). Sensor #1 exhibited significantly high selectivity toward NO_2_ (50 ppb) gas, with response and successive recovery at room temperature without any carrier gas flow or ultraviolet (UV) irradiation, whereas sensor #2 showed a high response and recovery toward SO_2_ (2 ppm) gas. Hicks et al. [41] presented a WO_3_ nanoparticle/water-soluble PANI particle composite doped on dodecylbenzenesulfonic acid at different mass ratios. The WO_3_/PANI nanocomposites were deposited onto interdigitated electrodes and characterized using electrochemical impedance spectroscopy in air and in acetone vapor. The nanocomposites showed increasing real impedance on exposure to acetone. However, the decreases in film resistance resulted from low WO_3_ nanoparticle loadings and low acetone concentrations. 

**Figure 4:**
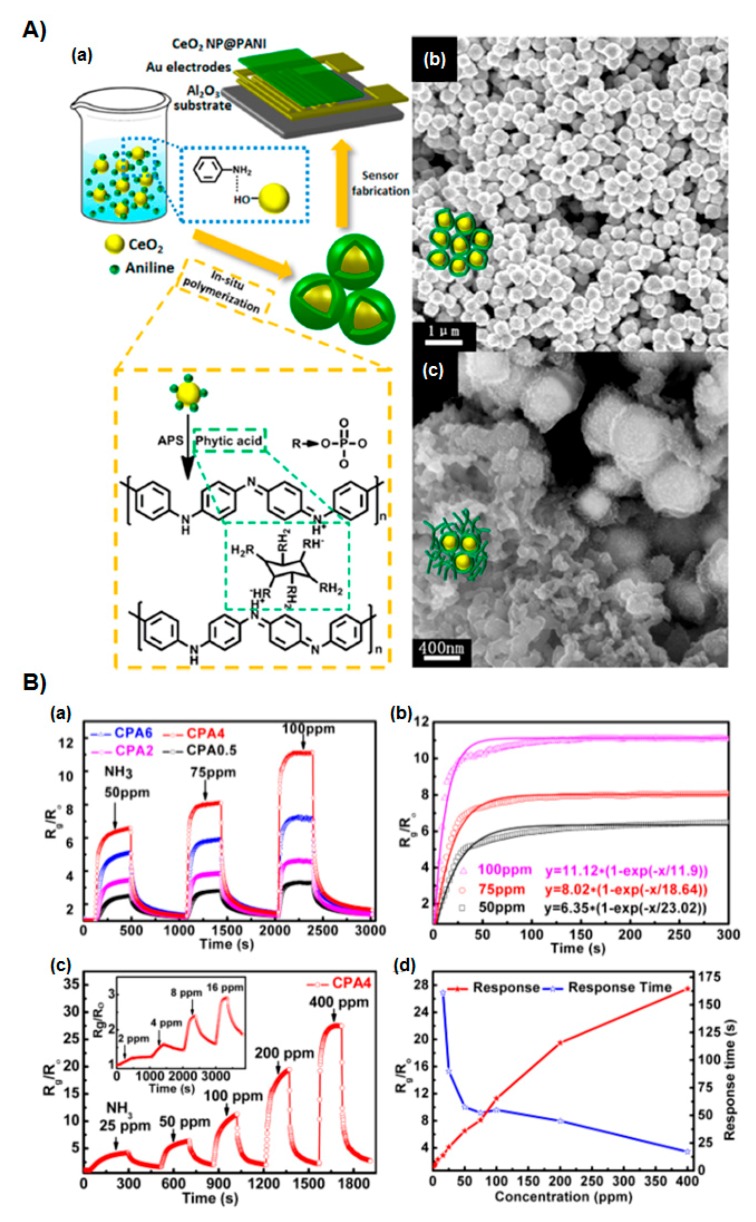
(**A**) (**a**) Schematics of the fabrication of PANI hydrogel/CeO_2_ core/shell gas sensors; (**b**) SEM image of CPA4 shows a well-defined core/shell structure in an optimal CeO_2_/aniline ratio; (**c**) SEM image of CPA0.5, which exhibits a three-dimensional network structure through a homogeneously grown PANI hydrogel. (**B**) (**a**) Response curves of CPA0.5 to CPA6 upon sequential exposure of 50, 75, and 100 ppm of NH_3_; (**b**) fitted curves (solid lines) and experimental results (scattered points) of CPA4 to 50, 75, and 100 ppm of NH_3_; (**c**) reversible and reproducible responses of CPA4 to different concentrations of NH_3_. Inset is the transient response of sequential exposure to 2, 4, 8, and 16 ppm of NH_3_. (**d**) Response and response time of CPA4 to various NH_3_ concentrations. The feed weight ratio of CeO_2_ to aniline was varied as 0.5, 2, 4, and 6, and the resulting composites were denoted as CPA0.5, CPA2, CPA4, and CPA6, respectively. Reprinted with permission from [43]. Copyright 2014, American Chemical Society.

This effect is caused by the balance of charge carriers in the film. The sensors can detect 10 ppm acetone vapor at room temperature. Talwar et al. [42] developed a new route for the synthesis of PANI nanofibers in the presence of various amounts of ZnO. The PANI nanofibers were prepared using a template technique, in which the ZnO nanoparticles were used as a template. The thick PANI films were deposited on an alumina substrate, and the sensing response to ammonia (NH_3_) gas was investigated. When the gas sensors were exposed to ammonia, PANI would change from the conducting emeraldine salt form to the insulating emeraldine base form via deprotonation. An optimal sensing response was obtained with PANI nanofibers in the presence of 30 wt % ZnO powder. Wang et al. [43] described a room-temperature NH_3_ gas sensor with high response and long-term stability, including CeO_2_ nanoparticles coated by cross-linked PANI hydrogel (CeO_2_@PANI, CPA). The core-shell nanocomposites were synthesized by in situ polymerization with different weight ratios of CeO_2_ nanoparticles and aniline. The core-shell nanohybrids demonstrated an increased response to NH_3_ concentration from 6.5 to 50 ppm at room temperature, which would be attributed to a p−n junction formed by the intimate contact between CeO_2_ and PANI (Figure 4). Furthermore, the stability was examined in the presence of phytic acid working as a gelator, which helped the PANI accommodate itself and enhance the mechanical strength and chemical stability of the sensors by preventing a “swelling effect” in high relative humidity (Figure 4Aa).

**Figure 5:**
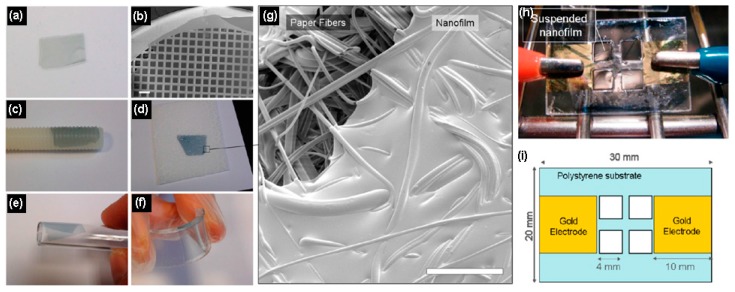
PEDOT:PSS/iron oxide nanoparticle composite nanofilms transferred to various substrates: (**a**) freestanding PEDOT:PSS/Nanoparticle 5 nanofilm (2 cm × 1 cm) floating in water after PVA dissolution. (**b**) SEM micrographs showing PEDOT:PSS/Nanoparticles 1 nanofilm collected onto a steel mesh (scale bar 100 μm). Nanofilms collected onto (**c**) Teflon screw, (**d**) paper, (**e**) plastic tube, and (**f**) flexible PDMS. (**g**) SEM image of the edge of a nanofilm collected on paper showing the paper fiber structure to which the nanofilm conforms (scale bar 50 μm). (**h**) A picture and (**i**) the structure of the samples used for humidity-sensing studies. The samples were referred to as PEDOT:PSS/Nanoparticles *x*, denoting films prepared using a colloidal solution with a concentration of *x* mg mL^−1^ iron oxide nanoparticles (*x* = 0, 1, 5). Reprinted with permission from [45]. Copyright 2013, American Chemical Society.

Zampetti et al. [44] demonstrated a hybrid sensor consisting of a nanofibrous layer of TiO_2_ coated with a thin film of PEDOT:polystyrene sulfonate (PEDOT:PSS), which was investigated as a potential sensing material for NO. The stoichiometric oxidation of NO into NO_2_ by CrO_3_ enabled the conductometric sensor to detect NO_2_ gas concentrations as low as 1 ppb, which is suitable for asthma monitoring. Taccola et al. [45] prepared free-standing nanocomposite ultrathin films based on PEDOT:PSS and embedding iron oxide nanoparticles. These nanocomposite films were fabricated by spin-coated assisted deposition in conjunction with a release technique to detach them from the substrate. The nanocomposite films could transfer onto substrates due to their high conformability, thus preserving their functionalities. The effect of the addition of iron oxide nanoparticles on the structural and functional properties of the PEDOT:PSS nanofilms was inspected through topography, thickness, magnetic and magneto-optical activity, and conductivity characterizations. PEDOT:PSS and PEDOT:PSS/iron oxide nanoparticle nanofilms were investigated as resistive humidity sensors. Their response to humidity was found to increase with increasing nanoparticle concentration (Figure 5).

**Figure 6:**
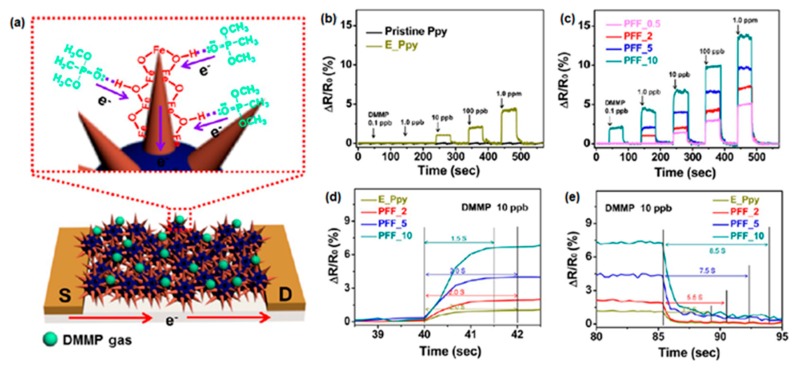
(**a**) DMMP gas detection mechanism of hybrid FeOOH nanoneedles on the PPy nanoparticles at room temperature. Reversible and reproducible responses are measured at a constant current value (10^−6^ A) with various amounts and types of FeOOH on the PPy surfaces. Normalized resistance changes upon sequential exposure to various DMMP concentrations of (**a**) pristine (black), E_PPy (yellow), and (**b**) hybrid PFF nanoparticles (pink, PFF_0.5; red, PFF_2; blue, PFF_5; green, PFF_10). The (**c**) response and (**d**) recovery times of hybrid PPy nanoparticles (yellow, E_PPy; red, PFF_2; blue, PFF_5; green, PFF_10) at 10 ppb of DMMP gas at room temperature. (**e**) The PFFs with 0.5, 2.0, 5.0, and 10.0 wt % FeOOH precursors are denoted as PFF_0.5, PFF_2, PFF_5, and PFF_10, respectively. Reprinted with permission from [47]. Copyright 2013, American Chemical Society.

Shin et al. [46] presented a sensor of toxic NO_2_ gas at room temperature based on multidimensional porous Fe_2_O_3_ nanorod-decorated carbon nanoparticles (MPFCNPs), which were fabricated using a dual-nozzle electrospray, thermal stirring, and heat treatment. PPy nanoparticles with FeOOH nanorods were synthesized using electrospun Fe^3+^ ions, which were adsorbed on the PPy nanoparticle surface followed by thermal stirring to grow nanorods without aggregation, and were then fabricated through heat treatment, with the porous structure created in the Fe_2_O_3_ nanorods by hydroxyl group decomposition. The response of the MPFCNP sensor was highly sensitive (1 ppm). Furthermore, sensitivity improved with decreasing diameters of the MPFCNPs and increasing Fe_2_O_3_ nanorod lengths. Lee et al. [47] described multidimensional FeOOH nanoneedle-decorated hybrid PPy nanoparticles (PFFs), which were synthesized by a dual nozzle electrospray and heat stirring process. To decorate FeOOH nanoneedles on the PPy surface, FeOOH particle-decorated PPys (E-PPy) were fabricated as starting materials. The E-PPy particles were arranged by dual-nozzle electrospray because Fe^3+^ dispersed on the surface reacted with OH^−^ in the collector solution without the aggregation of each particle. The hybrid PPFs detected a nerve gas agent, DMMP, at room temperature with high sensitivity. The minimum detection level of PFFs was as low as 0.1 ppb, which was higher than that of other hybrid materials (Figure 6).

Xiang et al. [48] prepared a composite of PPy and graphene nanoplates (GNs) decorated with TiO_2_ nanoparticles (TiO_2_@PPy-GN) by a sol–gel process combined with in situ chemical polymerization. The TiO_2_@PPy-GN nanocomposites demonstrated a good response to NH_3_ gas at room temperature, which could be attributed to the formation of a p−n junction between TiO_2_ and PPy-GN. Moreover, the TiO_2_@PPy-GN thin film exhibited enhanced NH_3_ sensing properties, higher sensitivity and much faster response than PPy-GN under the same conditions. Lee et al. [49] presented urchin-like PPy nanoparticles with different diameters, which were synthesized by a dual-nozzle electrospray and vapor deposition polymerization method. The urchin-like PPy nanoparticles were tested in various hazardous chemical gas sensors at room temperature, and they efficiently exhibited a greater sensitivity for NH_3_ due to their large surface area and 10 to 100 times higher minimum detectable levels of common analytes.

Huang et al. [50] prepared PTh/WO_3_ organic–inorganic hybrids, which were synthesized by an in situ chemical oxidative polymerization technique. The PTh/WO_3_ hybrids exhibited higher thermal stability than pure PTh and demonstrated a high response and good selectivity for detecting NO_2_ gas at ppm levels, and the 10 wt % PTh/WO_3_ hybrid exhibited the highest response at 70 °C. Xu et al. [51] reported SnO_2_ hollow sphere/PTh hybrid materials that were fabricated by an in situ chemical oxidative polymerization method. The SnO_2_ hollow sphere/PTh hybrids demonstrated a powerful synergistic interaction between the SnO_2_ hollow sphere and PTh, and the hybrids had higher thermal stability than pure PTh, showing high selectivity, good response, and a comparatively short recovery time to NO_2_ gas at 90 °C. The enhanced gas performance was due to the high surface area of the hybrids and the p–n heterojunction arranged between p-type PTh and n-type SnO_2_ hollow spheres. Guo et al. [52] prepared PTh/nanosized WO_3_ organic–inorganic hybrids fabricated by a chemical oxidative polymerization and colloidal chemical method. The PTh/WO_3_ hybrids with different mass fractions of PTh were acquired by mechanically mixing the prepared PTh and WO_3_. The hybrid composite exhibited higher response for NO_2_ sensing at <90 °C than pure PTh and WO_3_ sensors. The response of the PTh/WO_3_ hybrids was influenced by the PTh mass fraction. The 20 wt % PTh/WO_3_ hybrid demonstrated high response and good selectivity to NO_2_ gas at <90 °C. 

#### 3.1.2. Metal Hybrids

Patil et al. [53] prepared a thin film of Cu nanoparticles intercalated with PANI nanocomposites deposited by in situ oxidative polymerization of aniline in the presence of various concentrations of Cu nanoparticles at room temperature. The nanocomposite thin films and pure PANI exhibited a selective response toward NH_3_, but the nanocomposite thin films improved the sensor response and response kinetics. The response and recovery time of the nanocomposite film were 50 ppm of NH_3_ and 7 s, respectively. Moreover, the nanocomposite films could reversibly detect NH_3_ concentrations of 1 ppm or less. Cho et al. [54] reported Pd-decorated nanoporous poly(aniline-*co*-aniline-2-sunfonic acid):poly(4-styrenesulfonic acid) (P(ANI-*co*-ASA):PSS) nanostructures fabricated by a novel method and demonstrated the sensing response to hydrogen (H_2_) gas. Both P(ANI-*co*-ASA) and PSS supplied sufficient sulfonic acid (–SO_3_H) groups to efficiently anchor Pd nanoparticles, which resulted in enhanced interactions with H_2_ gas. The pores were 10−30 nm in diameter, and the enlarged effective surface area enabled interaction with H_2_ gas, resulting in much higher sensitivity compared to the non-porous counterpart. The porous Pd-decorated P(ANI-*co*-ASA):PSS nanocomposites achieved a detection limit of 5 ppm for H_2_ gas at room temperature. Bai et al. [55] presented PANI/Ag nanowire composites fabricated by chemically polymerizing PANI on top of dispersed Ag nanowires. The PANI/Ag nanowire composite surfaces were flexible and optically transparent, and they showed a sensing response to NH_3_. Organizing the PANI/Ag nanowire composite surface as chemiresistors led to resistance measurements that could detect 10 ppm of NH_3_ with an increase in the resistance. These PANI/Ag nanowire composites demonstrated selective sensing of NH_3_ versus ethanol, acetone, and n-propanol. Liu et al. [56] described a chemiresistive sensor based on PANI nanofibers decorated with Au nanoparticles, which was demonstrated to detect volatile sulfur compounds of human breath. The PANI nanofibers horizontally orientated on the insulating gap region of an interdigitated electrode were fabricated by template-free electrochemical polymerization. The decoration of the Au nanoparticles on the PANI nanofibers was made through the redox reaction between chloroauric acid and PANI emeraldine form. The fabricated Au/PANI gas sensor electrodes showed excellent sensing response to H_2_S (<1 ppm) and CH_3_SH (<1.5 ppm) gases. The sensing ability of the fabricated Au/PANI electrode on volatile sulfur compounds contained in human breath was proved by their response upon exposure to the exhaled breath of a healthy volunteer after ingesting raw garlic.

**Figure 7:**
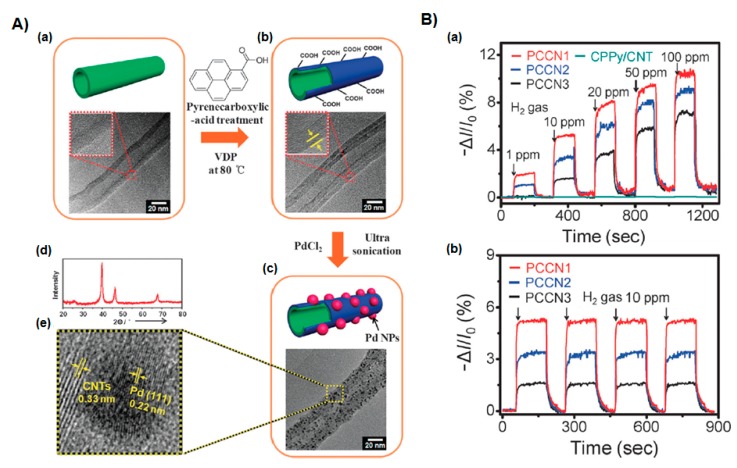
(**A**) Synthesis process of the PCCNs: (**a**) Bare CNT (inset indicates HR-TEM image of CNT of 30 nm in diameter); (**b**) ultrathin CPPy layer-wrapped CNT (inset indicates HR-TEM image of CPPy-CNTs with the skin thickness of 5 nm in diameter); (**c**) PCCNs; (**d**) XRD pattern; (**e**) HR-TEM of Pd nanoparticles. (**B**) Sensing performance of H_2_ sensors based on the nanohybrids. Real-time responses of the nanohybrid sensors (**a**) upon cyclic exposure to H_2_ (1 to 100 ppm, *V*_ds_ = −50 mV) and (**b**) on periodic exposure to 10 ppm H_2_ (Δ*I*/*I*_0_ = (*I* − *I*_0_)/*I*_0_, where *I*_0_ is the initial current, and *I* is the instantaneous current). Reprinted with permission from [61]. Copyright 2013, Royal Society of Chemistry.

Lee et al. [31] fabricated porous Pd-coated carboxylated PPy (CPPy) nanoparticles that detected hydrogen gas. The Pd/CPPy nanoparticles were developed through alkyl functionalization of CPPy nanoparticles using alkyl amine chain, followed by chemical reduction of palladium precursor (PdCl_2_) in the solution. The Pd/CPPy nanoparticles showed a detection limit of 0.1 ppm at room temperature, which is 10 to 100 times more sensitive than other nanocomposite-based chemical sensors. Tiwari et al. [57] presented Cu phthalocyanine-incorporated PPy synthesized by electropolymerization. The composite was utilized for the sensing of a nerve gas simulant DMMP and other vapors, including methanol, ethanol, benzene, toluene and hexane, which effectively exhibited greater selectivity for DMMP than other vapors. Hong et al. [58] prepared a Pd/PPy nanocomposite fabricated using a facile method including thermal dynamic refluxing of Pd salt and encapsulation of Pd nanoclusters with PPy by vapor phase polymerization. The nanocomposite film demonstrated a sensing response toward NH_3_ gas of 50–2000 ppm at room temperature, which is higher than pure PPy. Additionally, the electrical response was fast, reversible, and reproducible, with response and recovery times of 14 and 148 s, respectively, for 1000 ppm NH_3_ gas. The nanocomposite depended on the size of Pd nanoparticles and the morphology of the nanocomposite film. Gaikwad et al. [59] reported a facile synthesis of Pt/PPy nanocomposites, which were fabricated using in situ reduction of [H_3_O]_2_[PtCl_6_] by the amine group in the pyrrole monomer and oxidation of pyrrole to form PPy. The reactions were carried out at various temperatures to alter the degree of reduction of platinum precursor and doping of PPy with a Pt(II) chloro complex. The fabricated Pt/PPy nanocomposite gas sensors were investigated for their sensing response to liquefied petroleum gas (LPG), which resulted in good sensing at a relatively low temperature. Zhang et al. [60] reported a one-pot method that was used to fabricate Au/PPy nanocomposites, in which 4.2 nm Au nanoparticles were uniformly deposited on PPy. Control experiments revealed that lysine plays an important role in the synthesis of homogeneous Au/PPy nanocomposites. The Au/PPy nanocomposites exhibited great potential for detecting NH_3_ gas at room temperature, showing increasing sensor performance compared to pure PPy as a result of the functionalization of Au nanoparticles on PPy. Park et al. [61] described a novel method for designing an ultrathin CPPy layer-wrapped carbon nanotube (CNT) hybrid, which was fabricated by vapor deposition polymerization, and then mono-disperse Pd nanoparticles were immobilized on the PPy-CNT hybrid surface by a sonochemical method of PdCl_2_ (Figure 7A). The Pd-CPPy-CNT hybrids (PCCNs), were integrated as transducers in a field-effect transistor (FET) system. Field-induced sensitivity was observed, leading to a high-performance H_2_ gas sensor at low concentration (1 ppm) at room temperature. Three types of ultrathin CPPy skins were fabricated to control the amount of Pd nanoparticles; the pyrrole-3-carboxylic acid molar ratios were 1:15 (PCCN1), 1:30 (PCCN2), and 1:60 (PCCN3). The population of Pd nanoparticles on the nanohybrid surface increased in the order of PCCN3 < PCCN2 < PCCN1. The size distribution of the controlled Pd nanoparticles was also proven, enabling the modulation of the H_2_ gas sensor performance, and the PCCNs demonstrated excellent reproducible and reversible responses (Figure 7B).

Park et al. [62] reported Ag nanoparticle-decorated PEDOT nanotubes, which were fabricated utilizing Ag ion reduction-mediated vapor deposition polymerization via one-pot synthesis because PEDOT can reduce Ag ions to Ag nanoparticles. Ag/PEDOT nanotubes showed efficient NH_3_ gas sensing due to a high surface area and improved conductivity, originating from oxidation PEDOT by Ag nanoparticles. Moreover, the small size and homogeneous distribution of the Ag nanoparticles allowed the Ag/PEDOT nanotubes to be reversible and reproducible. That study estimated the increasing concentration of AgNO_3_ in Ag/PEDOT nanotubes to discover the optimal concentration for NH_3_ gas sensing. The Ag nanoparticle concentration increased and passed a specific threshold amount, above which they aggregated and decreased the gas-sensing performance.

Zhao et al. [63] presented highly conductive PTh films prepared using a facile aqueous solution-processing technique with HAuCl_4_ as a doping agent. The density of free charge carriers was evaluated to be 4.48 × 10^21^ cm^−3^. Therefore, the color of the polymer films changed as main absorption bands bathochromically shifted from the visible to the near-infrared region. The Au^3+^ ion doped PTh films were easily dedoped when exposed to organic amines and thiol vapors. The colorimetric and chemoresistive changes during the dedoping process enabled dual mode logic-gate sensing of amine and thiol volatile vapors with high selectivity and a detection limit <1 ppm.

Table 1 lists other notable examples of CP inorganic hybrids-based sensors developed recently and summarizes their main sensing performance.

### 3.2. Organic-CP Hybrids

CPs have long been used as sensing materials for detecting vapor-phase molecules due to the change in resistivity/conductivity arising from the interaction between CPs and gas molecules. However, selective interaction, mechanical stability, and reversible adsorption/desorption toward gas molecules limited their application for efficient and reproducible sensing devices. Moreover, it is a challenging task to develop a sensor with high sensitivity, high selectivity and good repeatability for rapidly sensing molecules in vapor phase with humidity. To overcome the drawbacks, co-polymer hybrids based on the CPs have been explored for the specific interaction in gas sensing systems. In general, gas molecules act as electron donors or acceptors, leading to a change in electronic conductivity or resistance in chemiresistor gas sensors. The chemical functional group of the CPs can enhance the sensitivity and selectivity in electrochemical sensors, which can be readily introduced into CPs during polymerization with a functionalized monomer or post-treatment. Moreover, using two or more types of polymers could improve mechanical stability and implement a flexible sensor. 

Srinives et al. [64] introduced a primary amine-functionalized PANI thin film as a chemiresistor sensor for recognizing formaldehyde. Aldehydes are widely known for reacting with primary and secondary amines and producing Schiff bases and water by nucleophilic addition reaction under ambient conditions [65,66]. PPy and PANI are candidates as sensing materials for formaldehyde because of their amine-rich chemical structures. In 2005, a PPy thin film was reported to be a chemiresistor sensor for detecting formaldehyde [33]. However, the sensor showed low sensitivity because it had only secondary amine and a low surface-to-volume ratio due to the smooth compact thin film. To improve sensing performance, the CP was functionalized with strongly nucleophilic primary amine, promoting the nucleophilic addition reaction. Moreover, the morphology of the surface became more uneven after treatment, providing a larger surface area. The devices were modified with three kinds of primary amine: phenylenediamine (PDA) having a diamine with aromatic ring, l-lysine (LYS) having a diamine with alkyl backbone and poly-l-lysine (PLY) at room temperature. The treated PANI film with primary amines showed significantly enhanced sensitivity and selectivity compared to a pristine PANI film. In particular, the LYS-treated PANI film displayed the best sensor activity, with a lower detection limit of 400 ppb for aldehyde gas due to the nucleophilicity of amine. The result of LYS-treated PANI film presented far better sensing performance than PPy thin film and previous other sensors [67,68,69,70].

**Figure 8:**
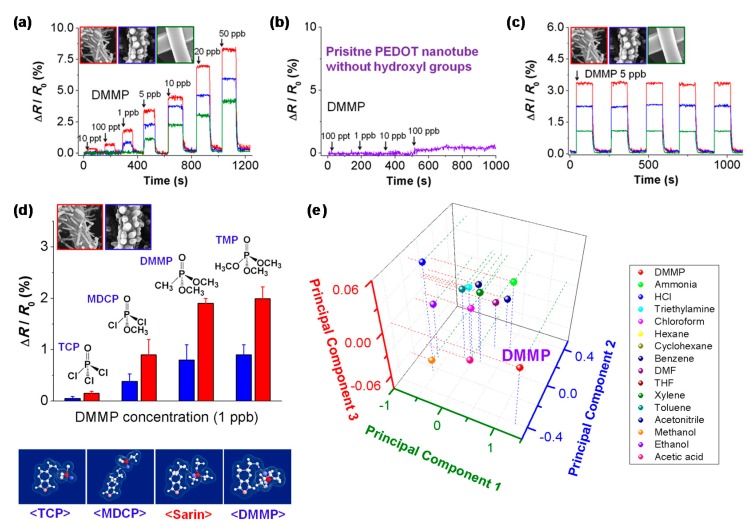
Dynamic sensing performance of HPEDOT nanotubes toward DMMP gas (inset; SEM images of prepared nanotubes with diverse morphologies in real time. (**a**) Sequential exposure to different DMMP gas concentrations (10 ppt to 50 ppb) in real time; (**b**) pristine PEDOT nanotube without hydroxyl groups; (**c**) periodic responses to 5 ppb DMMP; (**d**) selective response to similar organophosphorus molecules at 1 ppb (trichlorophosphate, TCP; methyl dichlorophosphate, MDCP; DMMP; trimethyl phosphate, TMP); (**e**) principal component analysis plots of the 16 vapors at 4 ppm. Reprinted with permission from [71]. Copyright 2012, American Chemical Society.

In 2012, Kwon et al. [71] demonstrated a high-performance DMMP gas sensor based on hydroxylated PEDOT (HPEDOT) nanotubes. DMMP is one of the nerve gas simulant organophosphate molecules that can induce serious damage in the nervous system. Many technical methods have been developed to detect nerve agents at low concentrations because they are odorless and colorless. The phosphoryl group of DMMP provides great strength in hydrogen-bond basicity, thus DMMP acts as a strong hydrogen bond base that accepts protons from the HPEDOT. Because PEDOT is a p-type semiconductor, this phenomenon is accompanied by a decrease in PEDOT conductivity via intermolecular hydrogen bonding. Figure 8 shows the dynamic response profiles of a HPEDOT sensor toward DMMP gas. Remarkably, this sensor exhibited a limit of detection of 10 ppt on exposure to DMMP vapors. By contrast, pure PEDOT nanotubes with no functionalized hydroxyl groups did not show a notable response at the same concentration, indicating that hydroxyl groups improve the sensing performance. The HPEDOT nanotubes had rapid response times (less than 1 s) and recovery times (3−25 s). In related work also published in 2016, Kwon et al. investigated the sensing responses with different functionalization degrees for DMMP gas sensors [6]. The sensing performance clearly depended on the amount of the functional group introduced. As a result, the sensitivity increased as the amount of carboxyl group increased. In addition, as a control, PPy nanotubes with no carboxyl groups showed measurable resistance changes only at high concentrations of more than 10 ppm. These results indicate that the carboxyl group plays a pivotal role in producing chemiresistive responses toward DMMP. 

Jun et al. [72] also reported the effect of introduced functional groups of co-polymers in chemical sensing performance. CPPy nanoparticles were prepared via copolymerization with two types of monomers, pyrrole and pyrrole-3-carboxylic acid, in an aqueous solution with polymer to prevent aggregation and metal cation complexes as the initiator [73]. Moreover, the amount of carboxyl groups was controlled with pyrrole monomer-to-pyrrole-3-carboxylic acid monomer weight ratios. Three types of C-PPy NPs with pyrrole monomer-topyrrole-3-carboxylic acid monomer weight ratios of 45:1 (C-PPy_1), 30:1 (C-PPy_2), and 15:1 (C-PPy_3) were prepared. Monodispersed CPPy nanoparticles containing different amounts of carboxyl groups were successfully fabricated, and carboxylic functional groups were confirmed by X-ray photoelectron spectroscopy (XPS) and Fourier transform infrared (FT-IR) measurements. Ammonia sensors based on C-PPy_1, C-PPy_2, and C-PPy_3 were measured in real time. The resistance of the sensors gradually increased after exposure to ammonia. They displayed enhanced sensitivity (detection limit, 0.1 ppm) as the ratio of carboxylic functional groups increased, demonstrating the effect of carboxyl groups in the ammonia sensor. This result is because the carboxylic groups on the surface of the PPy work as supplementary active sites toward ammonia gas by hydrogen bonding. 

In 2016, Manmatha et al. [74] reported a mechanically stable freestanding doped PANI/poly(vinyl alcohol) (PVA) composite membrane. It was prepared by oxidative polymerization of aniline in the presence of sodium dodecyl sulfate (SDS) at 0 °C under nitrogen atmosphere, and camphorsulfonic acid (CSA), l-aspartic acid (ASP) and p-toluenesulfonic acid (PTSA) were used as the doping agent during the synthesis of PANI. Membranes with thicknesses of 0.15–0.18 mm were obtained by a casting method of the blended solution with these doped CPs and PVA in dimethyl sulfoxide (DMSO). The doped polymer membrane showed good sensing performance and repeatability of the response towards aliphatic alcohols in 200 ppm of methanol vapor.

**Figure 9:**
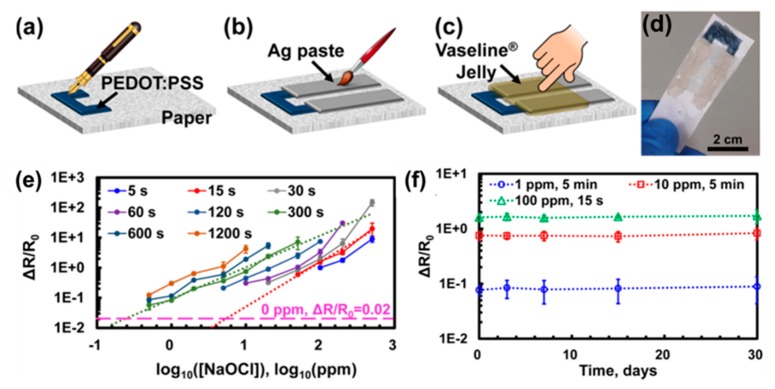
Schematic diagram of fabrication methods for paper-based free chlorine sensor strip based on PEDOT/PSS. (**a**) First, drawing of film as sensing active layer by using a pen filled with PEDOT/PSS solution; (**b**) second, drawing of electrodes with silver paste; (**c**) protecting the Ag electrodes from water with petroleum jelly; (**d**) photograph of manually prepared sensor kit; (**e**) sensing responses with different free-chlorine concentrations; (**f**) Storage stability. Reprinted with permission from [75]. Copyright 2016, American Chemical Society.

Qin et al. [75] reported an electrochemical free-chlorine sensor based on paper with PEDOT/PSS (Figure 9). Unfortunately, although PEDOT is a highly conductive polymer, it has limitations for various applications because it is not soluble by itself. Thus, it was reported that it could be embedded in PSS via the so-called Baytron-P process, providing a water-soluble PEDOT/PSS complex. The fabrication process is very easy and simple without any instruments or equipment. First, PEDOT/PSS solution was filled in the cartridge pen and used when drawing the sensing channel. Two electrodes connecting the PEDOT/PSS channel were then drawn with silver paste, and a petroleum jelly was manually applied to the silver electrodes to minimize the noise. The chlorine sensor based on the PEDOT/PSS channel was constructed without a professional technique. The sensor responses toward different free-chlorine concentrations were represented accurately at low concentrations in a short time because PEDOT was oxidized by free-chlorine quickly. Moreover, a wide sensing range (0.5–500 ppm) was obtained. To investigate reusability and storage stability, the sensor was stored in ambient air at room temperature and tested every few days for one month. The degradation of the sensor performance was less than 15% in 30 days, indicating excellent stability and reusability. 

Esteves et al. [76] illustrated chemiresistive gas sensors based on multi-electrode arrays modified with different porphyrin species. Among the several import factors, a highly sensitive and selective response toward specific analytes in several mixture gases is most important. In this paper, the authors introduced a new kind of poly(2-phenyl-1,4-xylylene) (PPPX) composite, doped with free-base porphyrins (meso-tetra(phenyl)porphyrin (H2TPP) [77], meso-tetra(2,3,4,5,6-pentafluorophenyl)porphyrin (H2TPFP) [78], 5,15-({3,5-bis(isopentyloxy)benzene}porphyrin (H2BTBOP) [79]) as a selective sensing material instead of organic Lewis acids. Prepared composites were deposited onto metallic interdigitated electrodes by spin-coating and exposed to four organic solvents, i.e., propanone, toluene, ethanol, and ethyl acetate, under 61% relative humidity. The response of the multi-electrode array was analyzed via a three-dimensional scatter plot composed by the relative resistance change of the three sensors toward four kinds of gases. The results of the resistance change were directly influenced by the interaction between the sensing materials and each solvent because distinct organic vapor led to different degrees of rotation of the polymeric chain, affecting the electrical conductivity. The scatter plot showed a unique pattern, providing the identification of four volatile organic solvents.

**Figure 10:**
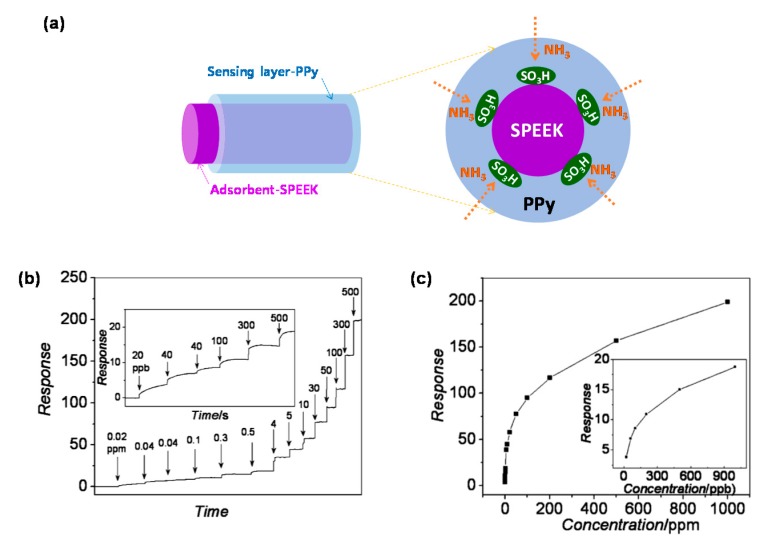
(**a**) Illustration of the SPEEK/PPy core-shell nanofibers for NH_3_ sensor; (**b**) real-time responses to NH_3_ in the range of 0.02−500 ppm (inset: the concentration range of 20−500 ppb); (**c**) the sensor response as a function of the NH_3_ concentration (inset: the response curve in the range of 20−1000 ppb). Reprinted with permission from [80]. Copyright 2012, American Chemical Society.

Wei et al. [80] presented sulfonated poly(ether ether ketone) (SPEEK)/PPy core/shell nanofiber for an ultrasensitive and flexible NH_3_ sensor (Figure 10). SPEEK was used as polymeric adsorbent, providing stable and distinct responses at a very low concentration of NH_3_ (20 ppb), which was as high as 3.8 (Figure 10b). By contrast, the polyacrylonitrile (PAN)/PPy nanofibers as reference material responded to NH_3_ at the limit of detection (over 5 ppm). In addition, the response intensity of PAN/PPy was less than 1/12 of that of the SPEEK/PPy (toward 10 ppm NH_3_). The SPEEK was prepared by electrospinning as a template and core, which produced outstanding mechanical flexibility. Moreover, excellent anti-interference performance was obtained by injecting 100 ppm of H_2_, CO, C_2_H_2_, CH_4_, methanol, ethanol, toluene, and NH_3_ serially into the test chamber at ambient temperature for practical application. 

### 3.3. CNT/Graphene-CP Hybrids

CP hybrids based on nanocarbons, including CNTs [81], carbon nanofibers, fullerenes, carbon nanofoams, and graphene/RGO, have been widely investigated and have exhibited excellent sensing performance [82]. We discuss hybrids based on CNT and graphene for chemical sensors in this section. Recently, CNT or graphene/CP composites have shown dramatic improvements in gas-sensing performance under ambient conditions. CNTs and graphene [83] have excellent physical and electrical properties, such as extremely high carrier mobility and capacity, high Young’s modulus, transparency, and thermal stability. Remarkably, they have been considered promising candidates for electrical gas-sensing materials [84,85,86]. They have shown a high signal-to-noise ratio due to the high quality of their crystal lattices and chemical structure, providing screen charge fluctuations. Based on these advantages, CNTs and graphene have been widely introduced into CP channels in electric transduced sensor systems. 

CNT and graphene provide a large surface area, leading to better signals because gas molecules can penetrate hybrids rapidly and allow more opportunities for reaction. Moreover, they enhance intrinsic charge transport due to high carrier mobility [87]. Generally, sensing materials were connected between two electrodes, and the charge transport occurred in the electric transduced sensor system. There are three possible means of charge transport because the electrodes are not connected by a single polymer chain or graphene sheet in the hybrid system. The first is hopping between polymer chains, and the second is hopping between polymer chains and graphene sheets. The last is hopping between the graphene-connected polymer chains. Graphene plays important roles in improving the charge transport because graphene has extremely high mobility compared to the CPs. Thus, the addition of graphene in the polymer matrix created an easier path for charge transport between electrodes, even in small amounts. Another possibility is suggested in terms of tunneling the charge carrier between the graphene as a highly conducting region and CP as a slightly insulating medium. The graphene provides a dominant path for efficient carrier migration through the channels, resulting in decreased time in the sensor system. Last, graphene could influence electrical signal transmission geometrically. 

Huang et al. [88] presented a sensitive ammonia gas sensor based on reduced graphene oxide (RGO)/PANI nanoparticles hybrids. RGO sheets allowed the nanoparticles to form uniformly without aggregation during polymerization. The morphology of the RGO/PANI hybrid was confirmed by TEM images, and the interaction between RGO and PANI nanoparticles was analyzed by the peak of polaron–π* transition with UV-visible spectroscopy. Furthermore, the surface area values of 32 and 22 m^2^ g^−1^ for RGO/PANI hybrids and PANI nanoparticles, respectively, suggest that a larger surface area of RGO/PANI hybrids can be obtained, which provides much better sensing performance because of the advantage in the adsorption of NH_3_ gas molecules. As a result, the RGO/PANI hybrid sensor showed enhanced sensing performance by factors of approximately 3.4 and 10.4 compared to those of the sensors based on bare PANI nanofibers and bare RGO sheets toward NH_3_ gas at 50 ppm. Although the response time was also improved, a longer recovery period was needed, and the resistance could not return to its initial state. This result reveals that positive synergetic effects have probably occurred between PANI nanoparticles and RGO sheets in terms of the intrinsic electronic properties and large surface area. In general, RGO also showed p-type behavior having hole-like charge carriers under ambient conditions owing to absorbed humidity or oxygen, similar to PANI. Because NH_3_ is an electron donor, it can lead to decreases in the number of charge carriers, resulting in increased resistance. When the PANI was de-doped due to NH_3_ gas, the charge carrier was reduced. Finally, the circuit networks completely depended on the RGO sheets, which were affected by the attached PANI nanoparticles directly. As a result, an approximately 30% change in resistance can be observed for this device, which is better than those of the devices based on bare RGO and PANI nanoparticles. 

**Figure 11:**
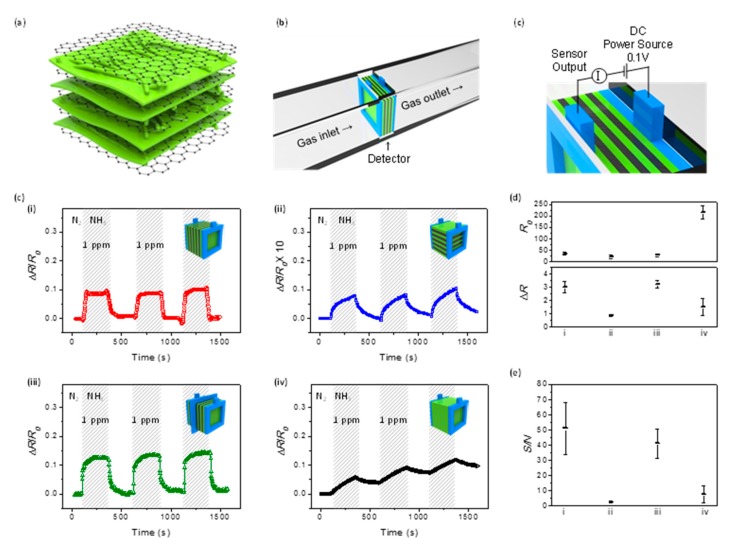
Schematic illustration of (**a**) G-*t*-PANI film; (**b**) gas flow and electrical setup for the chemical gas sensor; (**c**) sensing performance of a G-*t*-PANI film with various stacked orientation toward 1 ppm NH_3_ gas. ((**i**) a series type, (**ii**) a parallel type, (**iii**) a combination type connected with two series films of half-thickness, and (**iv**) a bulk *t*-PANI film); (**d**) initial (*R*_0_) and change (∆*R*) of resistance in resistometric sensor; (**e**) signal-to-noise ratio under the same conditions. Reprinted with permission from [89]. Copyright 2016, American Chemical Society.

Kim et al. [89] demonstrated the effect of a geometrical configuration with graphene/PANI (G-*t*-PANI) film in an electrical NH_3_ sensing system. The G-*t*-PANI architectures were prepared by ultrasonication after synthesizing PANI with a ternary dopant such as camphorsulfonic acid (CSA), dodecylbenzenesulfonic acid (DBSA), and D-sorbitol (SBT). As a result, the PANI was densely intercalated into the graphene sheets. Alternating layers of graphene and PANI were obtained, as shown in SEM images and analysis of elemental mapping. Moreover, the graphene was considered to preserve excellent electrical properties because no D peak was observed in Raman spectroscopy. The G-*t*-PANI film was integrated into the sensor device as a sensing channel (Figure 11). To observe the effect of the alternating configuration, a voltage was applied in perpendicular and parallel to the samples. In this experiment, four types of samples, i.e., a series type, a parallel type, a combination type connected with two series films of half-thickness and a bulk PANI film, as controls were constructed for an electrical NH_3_ gas sensor, and the response intensity as sensitivity and response/recovery time were profiled. As a result, the G-*t*-PANI exhibited markedly improved sensing performance compared to a bulk-PANI film. Interestingly, the series type showed a signal more than 10 times higher than the parallel type, and it had a significantly rapid response/recovery time. 

Li et al. [90] introduced graphene oxide/polypyrene (GO/PPr) composite films for sensors of volatile organic compounds (VOCs). PPr has potential as sensing material due to its strong fluorescence, high electrochemical conductivity, thermal stability, and lower toxicity. Despite its outstanding properties, no gas sensor transduced electrically based on it has been reported because the PPr is usually prepared as a powder. As a result, only PPr was impossible to apply to the active layers toward vapor sensors. In this article, GO/PPr composite film was fabricated via electrochemical co-deposition and characterized by SEM, FT-IR, TGA, and X-ray diffraction (XRD). PPr was successfully grown on GO sheets easily during polymerization due to the π−π stacking interaction between the GO sheet and the aromatic rings of PPr, and the chains of PPr layers played important roles in the interaction between GO sheets. As a result, the GO/PPr composite exhibited enhanced the mechanical properties and conductivity compared to powdery PPr film due to continuous structures, improving the junctions. Moreover, it showed thermal stability owing to the strong interfacial interaction. Finally, the VOC sensor based on GO/PPr showed high performance and a rapid, reversible response.

Abdulla et al. [91] presented an excellent ammonia gas sensor based on PANI-functionalized multiwalled CNTs (PANI-MWCNTs), which was synthesized by a simple and low-cost in situ chemical oxidative polymerization of aniline monomer on the surface of carboxylated multiwalled CNTs (C-MWCNTs). Prepared materials (C-MWCNTs and PANI-MWCNTs) were characterized by UV-visible spectroscopy, FT-IR spectroscopy, Raman spectroscopy, XPS, and high-resolution transmission electron microscopy (HR-TEM). The UV-visible spectra of PANI-MWCNTs displayed a very broad tailing-shaped band at approximately 700 nm, which revealed the highly doped form of PANI in the composite. A band at approximately 425−430 nm explained the doping electronic state by interactions between the imine sites of PANI and carboxyl groups in C-MWCNTs. Moreover, the composite showed more richness in quinoid units than PANI in FT-IR spectra, indicating enhanced conductivity by the strong interaction between PANI and C-MWCNT via π−π stacking. Interestingly, this effect was also observed in a previous study [92]. The electrical conductivities of PANI and PANI-MWCNT were measured and calculated to be 3.336 and 33.374 S cm^−1^, respectively. The reason for improved conductivity was suggested to be the dopant effect or charge transfer from the quinoid unit of PANI to the MWCNT, inducing electron delocalization. The comparative sensing performance based on the C-MWCNTs and PANI-MWCNTs were carried out in the range of a trace level (2−10 ppm) of NH_3_ gas. The response of the sensor based on PANI-MWCNTs (15.5%) exhibited a more enhanced result several times higher than that of C-MWCNTs (2.58%). Fast response and good reversibility were observed with PANI-MWCNTs compared to C-MWCNTs, attributed to the enhanced charge transfer. PANI-MWCNT composite was also introduced as a highly efficient CH_4_ gas sensor by Aldalbahi et al. [93]. Excellent sensing performance was observed at room temperature. Interestingly, the effect of the functional group of MWCNTs was studied with PANI/polymer/MWCNTs and PANI/polymer/MWCNTs-COOH nanocomposites in a CH_4_ gas sensor system. Both composites showed high sensitivity and selectivity toward CH_4_ gas. The sensor of PANI/polymer/MWCNTs exhibited a clear signal, quicker response time (<1 s), and higher sensitivity (3.1%) than those of the PANI/polymer/MWCNTs-COOH, which was attributed to the nonconductive property of the functional element. 

Sharma et al. [94] reported on PEDOT:PSS and PANI on MWCNTs, which were compared for their ammonia gas-sensing performance at room temperature, and the DC electric field and heating-cooling profile were controlled to optimize fast and complete recovery. Both sensors exhibited excellent sensitivity. However, upon comparison, the MWCNT/PEDOT:PSS composite was revealed to be considerably more sensitive (~16% better sensitivity) with a shorter response time (~15 min). Furthermore, the thermal stability of the MWCNT/PEDOT:PSS composite appeared to be much better than that of the MWCNT/PANI composite and showed excellent repeatability of standard resistance in the optimized recovery condition. 

Table 2 lists other notable examples of CP organic hybrid-based sensors developed recently, and summarizes their main sensing performance.

## 4. Conclusions

This review has introduced prominent findings for CP hybrids as chemo-electrical transducers, which can induce electrical sensing responses toward chemical gases, to aid in the understanding of recent trends in CP sensors. In terms of sensitivity, selectivity, and environmental stability, CP-based sensors still have room for improvement [13]. Several studies have demonstrated that versatile organic/inorganic CP hybrids can be fabricated using a metal/metal oxide, organic molecules, and nanocarbons as heterogeneous functional species, and they exhibited better sensing performance via synergistic effects such as enhanced sensitivity and outstanding chemical specificity. The benefits achieved by combining CPs with other materials into a hybrid can be summarized as follows:

(i) Metal oxides: Most metal oxides have finite bandgap energies, which can be coupled with those of CPs. As a result, judicious introduction of metal oxides can modulate the electrical/electronic properties of CPs, which often allows unique chemo-electrical transduction mechanisms. Additionally, metal oxides can be readily introduced as nanostructures in CPs, which leads to enhanced surface areas.

(ii) Metals: The conductivity of metals makes up for the low conductivity of CPs to prevent electrical signal loss. Some metals have a chemical affinity to specific gas species, where the metals act as chemical receptors to provide selectivity. Of course, metal nanostructures can also be introduced in CPs to enlarge the effective surface area that can interact with target gas species.

(iii) Polymers: Other kinds of polymers can be coupled with CPs to introduce chemical functional groups. The introduced functional groups contribute to the improvement of sensitivity and selectivity, and sometimes they can play an important role in modifying surface/interface properties of CPs.

(iv) Nanocarbons: The advantages of using nanocarbons include a well-defined, large surface area and a wide range of conductivity values. The carbon skeleton can also be modified with functional groups, which makes the nanocarbons more attractive.

In addition to these benefits, the chemo-electrical sensors based on CP hybrids have advantages such as room-temperature operation, tunable electrical/electronic properties, structural flexibility, and easy fabrication. CP-hybrid-based electrical sensing systems are still difficult to understand because many variables have a combined effect on the sensing performance of the hybrids, such as the independent/synergistic properties of each material component, the interfacial properties between the materials, the processing conditions, and environmental factors (e.g., temperature and humidity). To achieve further significant progress in CP-based sensor technologies, it is believed that in-depth research should be undertaken into what desirable physicochemical properties can be obtained when CPs are combined with other components, which is also dependent on the design, fabrication, and property control of new materials.

## Figures and Tables

**Table 1:** CP inorganic hybrid composites used in gas sensors.

CP	Hybrid material ^1^	Analyte	Response time	Sensing temperature ^2^	Detection limit	Ref.
PANI	ZnO	TEA	65−130 s	RT	10 ppm	[37]
	α-MoO_3_	TEA	35 s	RT	0.55 ppm	[38]
	Co_3_O_4_	CO	40 s	RT	75 ppm	[39]
	SnO_2_	SO_2_ and NO_2_	SO_2_: –, NO_2_: 5 min	RT	SO_2_: 2ppm and NO_2_: 50ppb	[40]
	WO_3_	Acetone	-	RT	10 ppm	[41]
	ZnO NPs	NH_3_	50 s	RT	25−100 ppm	[42]
	CeO_2_ NPs	NH_3_	57.6 s	RT	6.5−50 ppm	[43]
	Cu NPs	NH_3_	72 s	RT	1−50 ppm	[53]
	Pd NPs	H_2_	<90 s	RT	5 ppm	[54]
	Ag NWs	NH_3_	-	RT	5 ppm	[55]
	Au NPs	H_2_S and CH_3_SH	-	RT	H_2_S: 1 ppm and CH_3_SH: 1.5 ppm	[56]
PPy	Fe_2_O_3_ nanorod	NO_2_	-	RT	1 ppm	[46]
	FeOOH	DMMP	1.5−2.0 s	RT	0.1 ppb	[47]
	Graphene-TiO_2_ NPs	NH_3_	36 s	RT	1 ppm	[48]
	FeOOH-Cu^2+^	NH_3_	<1 s	RT	0.01 ppm	[49]
	Pd NPs	H_2_	4.5−12.5 s	RT	0.1 ppm	[31]
	Pt	LPG	-	170 °C	40 ppm	[59]
	Au NPs	NH_3_	-	RT	100 ppm	[60]
	Pd NPs	H_2_	<1 s	RT	1 ppm	[61]
PEDOT	TiO_2_ nanofiber	NO_2_	5−17 min	RT	1 ppb	[44]
	Fe_3_O_4_/γ-Fe_2_O_3_ NPs	humidity	1 s	RT	relative humidity 30−70%	[45]
	Ag NPs	NH_3_	2−3 s	RT	1 ppm	[62]
PTh	WO_3_	NO_2_	-	40–90 °C	10−100 ppm	[50]
	SnO_2_	NO_2_	-	90 °C	10−100 ppm	[51]
	WO_3_	NO_2_	-	RT–90 °C	-	[52]
	HAuCl_4_	amines and thiols	-	-	1 ppm	[63]

^1^ NP and NW note nanoparticle and nanowire, respectively; ^2^ RT indicates room temperature.

**Table 2:** CP organic hybrid composites used in gas sensors.

CP	Hybrid material ^1^	Analyte	Response time	Sensing temperature ^2^	Detection limit	Ref.
PANI	RGO	NH_3_	-	RT	5−50 ppm	[88]
	PVA	Aliphatic alcohols	4.6−7.8 s	RT	-	[74]
	Poly-3-hydroxybutirrate	NH_3_	100 s	RT	834 ppb	[95]
	HEC, PAA	H_2_O_2_	-	RT	0.5 mM	[96]
	PDA	Formaldehyde	-	RT	750 ppb	[64]
	polymer/MWCNT	CH_4_	<5 s	RT−60 °C	5–15 ppm	[93]
	MWCNT	NH_3_	< 6 s	RT	2–10 ppm	[91]
	Graphene	sugar	4 min	RT	D-fructose: 2.92 mM and D-glucose: 3.46 mM	[97]
	Graphene	NH_3_	<40 s	RT	1 ppm	[89]
PPy	Phthalocyanine	NH_3_	4−10 min	RT	25–90 ppm	[98]
	PPy-COOH NTs	DMMP	-	RT	0.5 ppb	[6]
	PPy-COOH NPs	NH_3_	2.0−3.5 s	RT	0.1 ppm	[72]
PEDOT	PSS	Chlorine	15−300 s	RT	0.5 ppm	[75]
	SWCNT	VOCs	-	RT	Methanol: 1.3%, Ethanol: 5.95%, methylethylketone: 3%	[99]
	PEDOT-OH NTs	DMMP	-	RT	10 ppt	[71]
	MWCNT	NH_3_	<15 min	RT	1 ppm	[94]
PT	Graphene	NH_3_	-	RT	250 ppb	[100]
PCDTBT	PCDTBT	NO	300 s	RT	5 ppm	[101]

^1^ NP and NT note nanoparticle and nanotube, respectively; ^2^ RT indicates room temperature.
